# Numerical Simulation of S-Shaped Current–Voltage Curves Induced by Electron Traps in Inverted Organic Photovoltaics

**DOI:** 10.3390/ijms23042039

**Published:** 2022-02-12

**Authors:** Shanglin Luo, Mingfang Huo, Qin Xue, Guohua Xie

**Affiliations:** 1 Department of Physical Science and Technology, Central China Normal University, Wuhan 430079, China; s2019112102@mails.ccnu.edu.cn (S.L.); mingfanghuo20@mails.ccnu.edu.cn (M.H.); 2 Sauvage Center for Molecular Sciences, Hubei Key Lab on Organic and Polymeric Optoelectronics Materials, Department of Chemistry, Wuhan University, Wuhan 430072, China

**Keywords:** organic photovoltaics, current–voltage, electron traps, numerical simulation

## Abstract

Organic photovoltaics (OPVs) differ from their inorganic counterparts because of inevitable electronic disorders and structural heterogeneity. Charge carrier traps are inevitable in organic semiconductors. A common failure mechanism of OPVs is the development of an S-shaped current density–voltage characteristic (*J-V* curve). Herein, we focus on investigating the underlying physical mechanism of S-shaped deformation of *J-V* curve of the inverted organic photovoltaic devices with bulk-heterojunction, proven by experiments with the n-doped electron extraction layer and numerical simulations assuming electron traps (0.1 eV deeper) in the electron extraction layer. The numerical simulations are quite consistent with the experimental results. In addition, the open circuit voltage induced by S-kink is exemplified to be enhanced after removing the electron traps in the interlayer by introducing a dopant of cesium carbonate.

## 1. Introduction

Organic photovoltaics (OPVs) are attractive systems for harvesting photonic energy due to their potential for large-scale production of light-weight, flexible, and cost-effective thin film technologies [1,2,3,4]. By employing new materials or interface modification, the performances of OPVs have been improved steadily. As known, the device performance is directly associated with the current–voltage characteristics (*J-V* curves) obtained under illumination. Frequently, the *J-V* curves often show undesirable S-shaped deformation under illumination. This will lead to apparent decrease of fill factor (*FF*) and open-circuit voltage (*V_oc_*). Reduced surface recombination for majority carriers [5,6], imbalance of charge carrier mobilities [7,8,9], dipoles defects at the interface [10,11], and poor quality of the interlayers between the electrode and the active layers [12,13], have been attributed as possible sources. All these phenomena result in carrier accumulation inside the device, and thus redistribution of the electric field. 

In recent years, OPVs with the inverted structure have been extensively investigated by using the modified indium tin oxide (ITO) as cathode contact, due to the better long-term ambient stability compared with the regular ones [14]. The ITO cathode of the inverted OPVs is usually modified by n-type materials, such as, cesium carbonate (Cs_2_CO_3_) and conjugated polymer. For example, the inverted bulk-heterojunction (BHJ) photovoltaic device with an alcohol/water-soluble conjugated polymer, poly[(9,9-bis(3′-(*N*,*N*-dimethylamino)propyl)-2,7-fluorene)-alt-2,7-(9,9-dioctylfluorene)] (PFN), was frequently used as interlayer between the active layer and the cathode (ITO) [3]. However, an apparent S-shaped *J-V* curve was observed since the performance was very sensitive to the thickness of the poorly conductive PFN. It is supposed that the S-shape curves may be ascribed to imperfect contact caused by electron traps in PFN [15]. This is similar to that the devices containing the interfacial layers of ZnO or TiO_x_ were commonly dominated by electron traps in the interfacial layers [16,17,18]. However, investigating the effect of traps by means of electrical simulations is still not well addressed. In this contribution, we used an effective device model with the discrete mid-gap states to study the S-shape effect induced by electron traps in the interlayers between the cathode and the active layer. Besides, the wxAMPS-1D (analysis of microelectronic and photonic structures) program was used to solve the general set of equations, which described the basic phenomena in photovoltaic devices [19]. By using the proposed model, the S-shaped *J-V* characteristics could be qualitatively reproduced. The simulation results suggest that the discrete acceptor-like gap states can cause a dramatic change in the *J-V* curve, shaping the standard exponential diode curve to S-shape. Throughout this investigation, we obtained a comprehensive understanding of the underlying physics of traps induced S-shaped *J-V* curves. These findings are important and applicable to the emerging photovoltaic devices because traps are easily formed in the solution-processed devices. Furthermore, to suppress the intrinsic electron traps of PFN by trap-filling method, we introduced Cs_2_CO_3_ into the PFN interlayer, and consequently the S-shape curve was effectively eliminated, which led to the apparently improved *FF* and *V_oc_* of the solar cell. The shift of *V_oc_* observed in the experiment can also be rationally explained with the numerical model. 

## 2. Results

### 2.1. Device Performance

**Figure 1:**
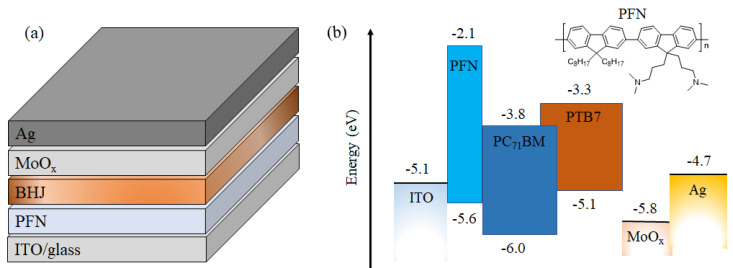
Schematic diagrams of the device structure (**a**) and energy level alignment along with the chemical structure of PFN (**b**). The energy levels of organic materials were mainly cited from [15].

**Figure 2:**
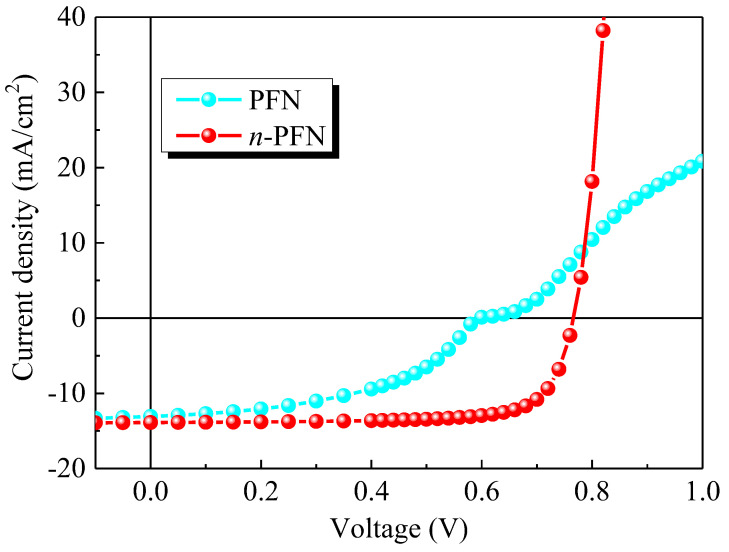
Measured *J-V* characteristics of the devices with the pristine PFN and Cs_2_CO_3_ doped PFN (*n*-PFN) under illumination.

**Figure 3:**
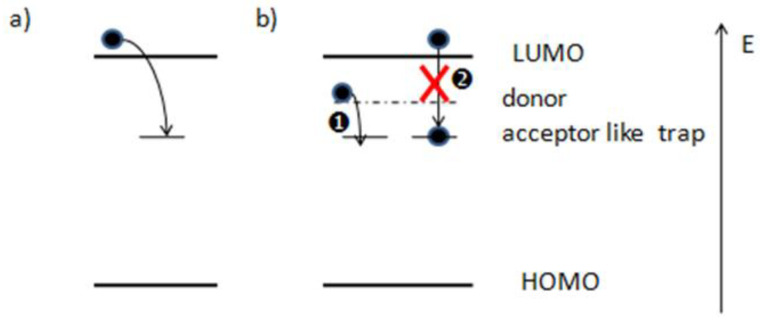
(**a**) Schematic diagram of trapping of an electron by a gap state. (**b**) Gap state filled by the additional dopant.

The experimental *J-V* curves of the devices (schematically shown in Figure 1) under illumination with the pristine PFN and Cs_2_CO_3_ doped PFN (*n*-PFN) are compared in Figure 2. The PFN interlayers with and without Cs_2_CO_3_ were very uniform (see Figure S1). However, the distinct S-shaped behaviors were observed in the devices with the pristine PFN (see Figure 2, Figures S2 and S3). After doping Cs_2_CO_3_ into the PFN layer, the *V_oc_* was increased from 0. 59 V to 0. 76 V, the *FF* from 49% to 76%, and the short-circuit current density (*J_sc_*) from 13.0 mA/cm^2^ to 13.9 mA/cm^2^. As the initial S-shape characteristics in our OPVs can be eliminated by adding Cs_2_CO_3_ into PFN, the S-shape *J-V* characteristics should originate from the PFN layer itself. It is known that traps can be either extrinsic or intrinsic. Intrinsic traps can arise from the energetic and structural disorders in organic semiconductors and are more difficult to be eliminated compared with extrinsic traps. Yan et al. found that trap density can be effectively decreased by trap-filling with 2,3,5,6-tetrafluoro-7,7,8,8-tetracyanoquinodim-ethane(F_4_-TCNQ) in the donor polymer [20]. Thus, we infer that, after introducing Cs_2_CO_3_ into PFN layer, the electron traps may be passivated by the addition of electrons introduced by molecular n-doping (see Figure 3) [21,22,23].

### 2.2. Numerical Model

In order to understand the electrical behavior in organic devices and interpret the experimental data, we need the flexible device models. The common device models based on a drift-diffusion approach have been successfully used to describe the electrical behavior of OPV [24,25,26]. Three coupled differential equations must be solved in a macroscopic simulation, which is based on the Poisson equation (see below).

(1) $$\frac{d^{2}\varphi }{dx^{2}}=\frac{q}{\varepsilon_{0}\varepsilon_{r}}\left[n(x)-p(x)+N_{A}^{-}(x)-N_{D}^{+}(x)+n_{t}(x)-p_{t}(x)\right]$$

where in the metal–insulator–metal model, $N_{A}^{-}\left(x\right)=N_{D}^{+}\left(x\right)=0$.

The drift diffusion equations respectively for the electrons and holes are listed below.

(2) $$-\frac{1}{q}\frac{dJ_{n}(x)}{dx}=-D_{n}\frac{d^{2}n(x)}{dx^{2}}-F\mu_{n}\frac{dn(x)}{dx}=G(x)-R(x,n,p)$$

(3) $$\frac{1}{q}\frac{dJ_{p}(x)}{dx}=-D_{p}\frac{d^{2}p(x)}{dx^{2}}+F\mu_{p}\frac{dp(x)}{dx}=G(x)-R(x,n,p)$$

In the above equations, q is the elementary charge, φ is the potential barrier, $\varepsilon_{0}$ is the permeability in free space, $\varepsilon_{r}$ is the relative dielectric permeability, *n* and *p* are the concentrations of the free electrons and holes, $n_{t}$ and $p_{t}$ are the concentrations of trapped electrons and holes, and $N_{D}^{+}$ and $N_{A}^{-}$ are the concentrations of the ionized donors and acceptors, respectively. $D_{n}$ and $D_{p}$ are the diffusion coefficients of electrons and holes, respectively. $J_{n}$ and $J_{p}$ are the electron and hole current densities. F denotes the electrical field, G and R are the generation and recombination rates of free charge carriers, and *μ_n_* and *μ_p_* are the charge carrier mobilities, respectively for electrons and holes. Here, we only consider Shockley–Read–Hall recombination. Therefore, we find that bimolecular recombination merely changes the S-Kink in the simulated *J-V* curves. 

The boundary condition for the electrical potential is described as below.

(4) $$\varphi \left(d\right)-\varphi \left(0\right)=V_{bi}-V$$

**Figure 4:**
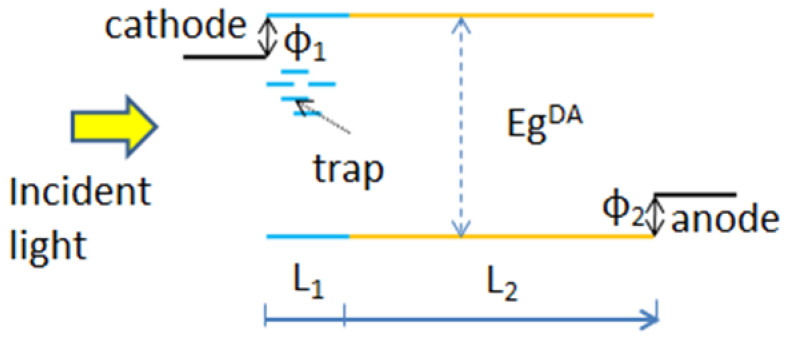
Schematic diagram of energy level alignment with electron traps and injection barriers used in the numerical simulation.

**Figure 5:**
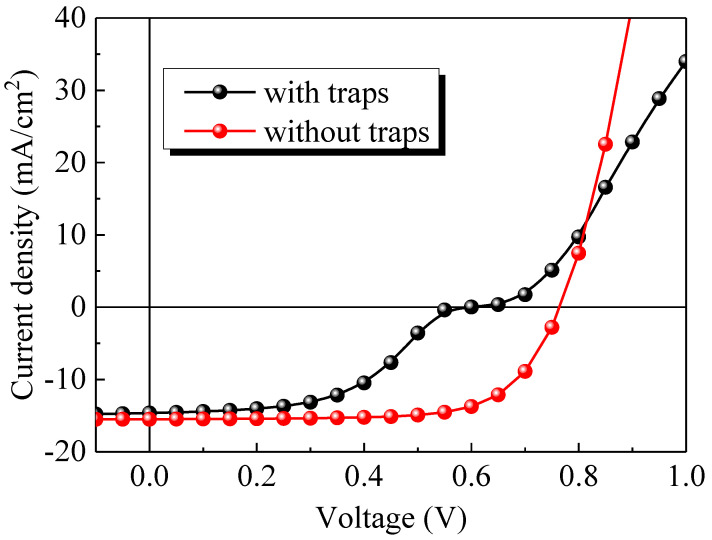
Simulated *J-V* characteristics of the devices with and without traps.

φ(0) and φ(d) are the energy barriers at the cathode contact and the anode contact, respectively. *V_bi_* is the built-in voltage and *V* is the applied voltage.

(5) $$n\left(0\right)=N_{c}\exp\left(-\frac{\varphi_{1}}{kT}\right)$$

(6) $$p\left(0\right)=N_{v}\exp\left(-\frac{E_{gap}-\varphi_{1}}{kT}\right)$$

(7) $$n\left(d\right)=N_{c}\exp\left(-\frac{E_{gap}-\varphi_{2}}{kT}\right)$$

(8) $$p\left(d\right)=N_{v}\exp\left(-\frac{\varphi_{2}}{kT}\right)$$

where $\varphi_{1}$ and $\varphi_{2}$ are injection barriers respectively for electrons and holes at the cathode and anode contacts. n(0) and p(0) represent the density of charge carriers at the interface of between the active layer and the interlayer. Meanwhile, n(d) and p(d) denotes the density of charge carriers at the interface between the active layer and the anode if no interlayer is introduced and d is the thickness of the active layer. $E_{gap}$ is the effective energy offset inside the active layer. $N_{c}$ and $N_{v}$ are the intrinsic densities of states at the conduction and valence bands, respectively. k is the Boltzmann constant and T is the absolute temperature. Using Equations (1)–(8), we can calculate the charge carrier concentrations and electrical potential. By integrating Equations (2) and (3), *J-V* curve under illumination or in the dark can be simulated. Here, we use the wxAMPS-1D program, an open software for solar-cell simulation software that is an enhanced version of AMPS-1D [27,28], to solve the above set of equations. WxAMPS-1D consists of the optimized algorithms, including Gummel and Newton methods, and the simulation stability is enhanced. Some of the parameters are adapted from the reference data available at the open source [29]. It is also possible to customize the data which are fit to the devices constructed in this investigation. For simplicity, we adapted the buffer layer for qualitative study of the S-shape effect in the *J-V* curves. The device structure diagram used in our simulation is shown in Figure 4, and the parameters used in the simulation are listed in Table 1. The numerically simulated *J-V* curves with the discrete acceptor-like gap states (a concentration of 10^18^ cm^−3^) and without any gap states in the buffer layer were illustrated in Figure 5. With the parameters given in Table 1, we retrieved a *V_oc_* of 0.59 V as for the device with traps, which was increased to 0.76 V as for the device without traps. The calculated *J_sc_* was slightly increased from 14.6 to 15.5 mA/cm^2^, and the *FF* increased from 49% to 70%, respectively, when no trapped electrons were involved in the electron extraction layer.

## 3. Discussion

**Figure 6:**
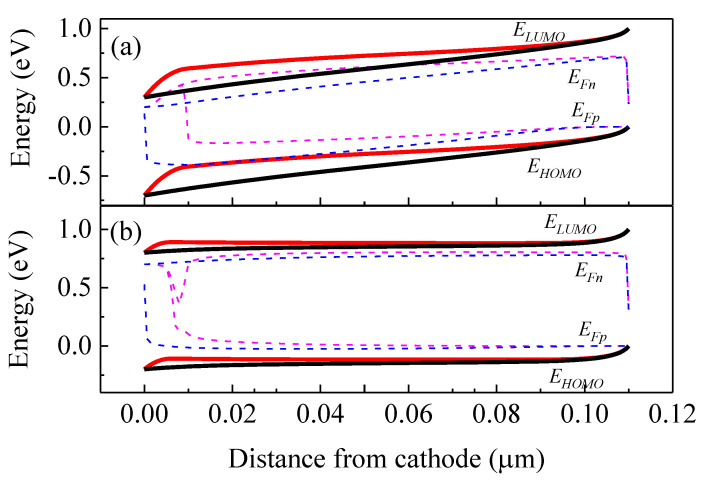
Energy distributions of the devices with/without traps (**a**) under 0.2 V and (**b**) under 0.7 V.

The deformation of the *J-V* curves originates from the internal energetic band structure and charge carrier distributions. The energetic band structures of the devices with/without acceptor-like defect states in the interlayer are shown in Figure 6a,b, respectively for the case under the applied voltages of 0.2 V and 0.7 V. The energy levels of the lowest unoccupied molecular orbital (LUMO) ($E_{LUMO}$) and the highest occupied molecular orbital HOMO ($E_{HOMO}$) of the device with traps are shown in thick solid red lines, while *E_LUMO_* and *E_HOMO_* of device without any traps are shown in thick solid black lines. The quasi-Fermi levels $E_{Fn}$ and $E_{Fp}$ of the device with traps are shown in thin dash magenta lines and those of the device without traps are indicated in thin dash blue lines. 

**Figure 7:**
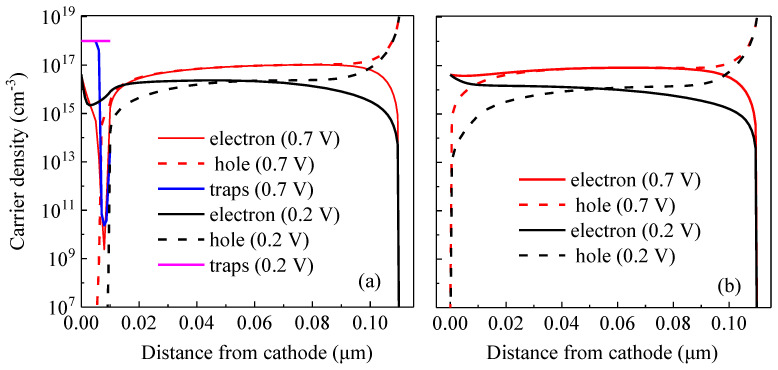
(**a**) Carrier densities of the device with traps at 0.2 V and 0.7 V. (**b**) Carrier distribution of the device without traps.

Figure 7a shows the carrier distribution of the device with traps at 0.2 V and 0.7 V, respectively. The black solid and dash lines indicate the electron and hole densities at 0.2 V, respectively. In contrast, the red solid and dash lines indicate the electron and hole densities at 0.7 V, respectively. Besides, the density of acceptor-like states that have trapped electrons are shown in magenta and blue lines respectively for the cases under 0.2 V and 0.7 V. For comparison, carrier distributions of the device without traps is shown in Figure 7b. As for the device with traps under the voltages below *V_oc_*, i.e., under extracting condition, the generated charges are extracted from the active layer into the electrodes, a region of the trapped space charge is formed near the cathode. As the applied voltage approaches to *V_oc_*, the effective internal voltage dropping over the trapped space charge region starts to decrease and thus this negatively charged region begins to narrow down. The rate of the traps to capture electrons is proportional to the number of free electrons, and the rate of the captured holes is proportional to the number of holes [30,31]. 

**Figure 8:**
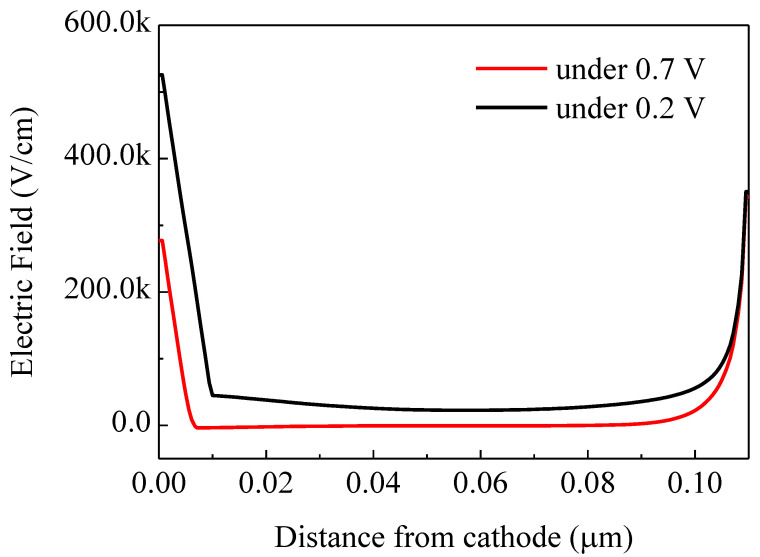
Electric field distribution of device with traps under 0.2 V and 0.7 V.

When the net rate of the captured holes is larger than that of electrons, the density of the acceptor-like states that have trapped electrons will decrease, which will cause the narrowed trapped space charge region. Therefore, we can observe that the hole density in the interlayer with traps under 0.7 V is increased while the electron density is reduced, in contrast to that of the device under 0.2 V, which is consistent with the narrowing of the trapped space charge region. At the interface between the active layer and the interlayer, fewer trapped space charges, fewer free electrons and more holes are observed. Consequently, the electric field across the interface between the active layer and the interlayer is reduced and will even change signs, as shown in Figure 8. The transition between both cases creates an S-kink around *V_oc_*.

**Figure 9:**
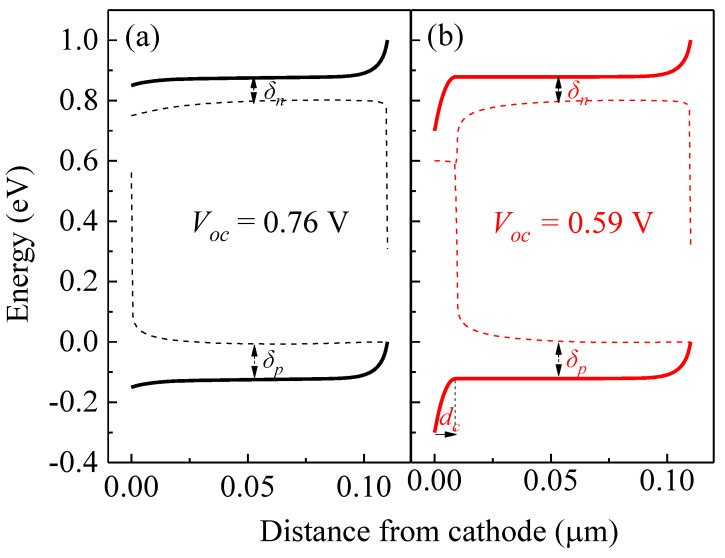
Calculated energy alignment referred to *V_oc_* of the devices (**a**) without and (**b**) with traps.

As shown in Figure 2 and Figure 5, the S-shape *J-V* curves lead to the loss of *V_oc_*. For the device without traps under the *V_oc_* of 0.76 V, the energy band diagrams are calculated and shown in Figure 9a. Moreover, Figure 9b illustrates the case of the device with traps.

Utilizing the metal–insulator–metal concept, we can express the $V_{OC}$ as below [32].

(9) $$qV_{OC}=E_{g}^{DA}-\max\left(\varphi_{2},\delta_{p}\right)-\max\left(\varphi_{1},\delta_{n}\right)$$

where $\delta_{n}=E_{LUMO}-E_{Fn}$ ($\delta_{p}=E_{Fp}-E_{HOMO}$) at x_max_ which corresponds to the case in the active layer where the product *np* is the largest. According to Figure 9, the $V_{OC}$ can be given by

(10) $$qV_{OC}=E_{g}^{DA}-\delta_{p}-\varphi_{1}$$

As in the case of the acceptor-like traps in the interlayer, the magnitude of the band bending from the immobile trapped space charge can be estimated as below.

(11) $$∆=\frac{q^{2}n_{t}d_{c}{}^{2}}{2\varepsilon_{0}\varepsilon_{r}}$$

where $n_{t}$ is the trap density mentioned previously, and $d_{c}$ is the thickness of the negatively charged region. Thus, the effective electron injection barrier ($\varphi_{1,eff}$) can be rewritten as $\varphi_{1,eff}=\varphi_{1}+∆$ and the effective built-in voltage ($V_{bi,eff}$) is changed to $V_{bi,eff}=qV_{bi}-∆/q$. From the energy band diagram of Figure 9b, we can estimate the thickness $d_{c}$ ≈ 8 nm. Therefore, according to Equation (11), ∆ ≈ 0.17 eV. Consequently, the shift of *V_oc_* between the devices with and without traps will be equal to −0.17 V, according to Equation (10), which is well consistent with the experiments. 

## 4. Materials and Methods

The device structure was ITO/PFN/PTB7:PC_71_BM/MoO_x_/Ag. The donor material PTB7—i.e., poly(thieno [3,4-*b*]-thiophene benzodithiophene)—and the acceptor material PC_71_BM, i.e., [6,6]-phenyl-C_71_-butyric acid methyl ester, were purchased from Lumtec and used as received. The interlayer material PFN (PLT105081B), purchased from Xi’an P-OLED Corp., was dissolved in methanol in the presence of a small amount of acetic acid (2 μL/mL) and its solution (2 mg/mL) was spin-coated on top of the prepatterned ITO substrate at 6000 r.p.m. for 10 min. As for the device with the so-called n-doping, PFN:Cs_2_CO_3_ (5:1, *w*/*w*) layer, ca. 10 nm, were spin-coated from the solution in methanol (2 mg/mL) at 6000 r.p.m. for 10 min. The PTB7:PC_71_BM (1:1.5, *w*/*w*) active blend layer (80 nm) was prepared by the solution (25 mg/mL) with the mixed solvent of chlorobenzene:1,8-Diiodoctane (97:3 by volume) spin-coated at 1200 r.p.m. for 2 min. A 10 nm-thick M_o_O_x_ and 50 nm-thick Ag were thermally evaporated, subsequently.

## 5. Conclusions

In summary, we investigated the trapped electrons of the interfacial layer in the inverted organic photovoltaics with the common bulk heterojunction, which contributed the S-shaped *J-V* curves verified by both experiments and numerical simulation. By analyzing the calculated energetic band structure and carrier distributions, we assign the changing of the trapped space charge region which will induce a change of the electric field distribution as the origin of the characteristic deformation. The S-kink led to the loss of *V_oc_* can be reasonably explained. A thorough understanding of the electron trapping effects is critical for designing high-performance organic photovoltaics where traps are very commonly found.

## Figures and Tables

**Table 1:** Parameters used in the simulation.

Parameter	Symbol	Numerical Value
Effective band gap	$E_{g}{}^{DA}$	1.2 eV
Electron mobility	$\mu_{n}$	$2\times 10^{-3}{\;\mathrm{cm}}^{2}V^{-1}s^{-1}\;\;$
Hole mobility	$\mu_{p}$	$2\times 10^{-4}{\;\mathrm{cm}}^{2}V^{-1}s^{-1}$
Relative static permittivity	$\varepsilon_{r}$	3.4
Injection barrier at cathode side	$\varphi_{1}$	0.1 eV
Injection barrier at anode side	$\varphi_{2}$	0 eV
Density of states at conduction band	$N_{C}$	$2\times 10^{18}\;{\mathrm{cm}}^{-3}$
Density of states at valence band	$N_{V}$	$2\times 10^{19}\;{\mathrm{cm}}^{-3}$
Distance to cathode	$L_{1}$	$10^{-6}\;\mathrm{cm}$
Distance to anode	$L_{2}$	$10^{-5}\;\mathrm{cm}$
Surface recombination velocity of electrons (cathode side)	$S_{n,cat}$	$10^{7}\;{\mathrm{cm}\;s}^{-1}$
Surface recombination velocity of holes (cathode side)	$S_{p,cat}$	$10^{7}{\;\mathrm{cm}\;s}^{-1}$
Surface recombination velocity of electrons (anode side)	$S_{n,an}$	$10^{7}{\;\mathrm{cm}\;s}^{-1}$
Surface recombination velocity of holes (anode side)	$S_{p,an}$	$10^{7}{\;\mathrm{cm}\;s}^{-1}$
Discrete acceptor-like gap state density	$n_{t,a}$	$10^{18}{\;\mathrm{cm}}^{-3}$
Capte cross section of electrons	$\sigma_{n},$	$10^{-10}{\;\mathrm{cm}}^{2}$
Capture cross section of holes	$\sigma_{p}$	$10^{-12}{\;\mathrm{cm}}^{2}$

## Data Availability

The data presented in this investigation is available in article or from the corresponding authors.

## Supplementary Materials

The following supporting information can be downloaded at: https://www.mdpi.com/article/10.3390/ijms23042039/s1.

## References

[1] Cui Y., Wang Y.M., Bergqvist J., Yao H.F., Hou J.H. (2019). Wide-gap non-fullerene acceptor enabling high-performance organic photovoltaic cells for indoor applications. Nat. Energy.

[2] Li G., Zhu R., Yang Y. (2012). Polymer solar cells. Nat. Photonics.

[3] He Z.C., Zhong C.M., Su S.J., Xu M., Wu H.B., Cao Y. (2012). Enhanced power-conversion efficiency in polymer solar cells using an inverted device structure. Nat. Photonics.

[4] Lin Y.B., Adilbekova B., Firdaus Y., Yengel E., Faber H., Sajjad M., Zheng X.P., Yarali E., Seitkhan A., Bakr O.M. (2019). 17% Efficient organic solar cells based on liquid exfoliated WS2 as a replacement for PEDOT:PSS. Adv. Mater..

[5] Wagenpfahl A., Rauh D., Binder M., Deibel C., Dyakonov V. (2010). S-shaped current-voltage characteristics of organic solar devices. Phys. Rev. B..

[6] Finck B.Y., Schwartz B.J. (2013). Polymer/fullerene photovoltaics from drift-diffusion simulations. Appl. Phys. Lett..

[7] Nelson J., Kirkpatrick J., Ravirajan P. (2004). Factors limiting the efficiency of molecular photovoltaic devices. Phys. Rev. B.

[8] Mihailetchi V.D., Wildeman J., Blom P.W.M. (2005). Space-charge limited photocurrent. Phys. Rev. Lett..

[9] Tress W., Petrich A., Hummert M., Hein M., Leo K., Riede M. (2011). lmbalanced mobilities causing S-shaped IV curves in planar heterojunction organic solar cells. Appl. Phys. Lett..

[10] Schulze K., Uhrich C., Schuppel R., Leo K., Pfeiffer M., Brier E., Reinold E., Bauerle P. (2006). Efficient vacuum-deposited organic solar cells based on a new low-bandgap oligothiophene and fullerene C60. Adv. Mater..

[11] Uhrich C., Schueppel R., Petrich A., Pfeiffer M., Leo K., Brier E., Kilickiran P., Baeuerle P. (2010). Organic thin-film photovoltaic cells based on oligothiophenes with reduced bandgap. Adv. Funct. Mater..

[12] Sharma A., Franklin J.B., Singh B., Andersson G.G., Lewis D.A. (2015). Electronic and chemical properties of ZnO in inverted organic photovoltaic devices. Org. Electron..

[13] Kim C.S., Lee S.S., Gomez E.D., Kim J.B., Loo Y.L. (2009). Transient photovoltaic behavior of air-stable, inverted organic solar cells with solution-processed electron transport layer. Appl. Phys. Lett..

[14] Tran H.N., Dao D.Q., Yoon Y.J., Shin Y.S., Choi J.S., Kim J.Y., Cho S. (2021). Inverted polymer solar cells with annealing-free solution-processable NiO. Small.

[15] Li Z., Guo J., Lu F., Liu C., Zhang X., Shen L., Wu H., Guo W. (2018). Eliminating light soaking effect of inverted polymer solar cells functionalized with a conjugated macroelectrolyte electron-collecting interlayer. Electrochim. Acta.

[16] Schmidt H., Zilberberg K., Schmale S., Fluegge H., Riedl T., Kowalsky W. (2010). Transient characteristics of inverted polymer solar cells using titaniumoxide interlayers. Appl. Phys. Lett..

[17] Romero B., Pozo G.D., Destouesse E., Chambon S., Arredondo B. (2014). Circuital modelling of S-shape removal in the current-voltage characteristic of TiOx inverted organic solar cells through white-light soaking. Organic. Electronics.

[18] Zhang D., Choy W.C.H., Xie F.X., Sha W.E.I., Li X.C., Ding B.F., Zhang K., Huang F., Cao Y. (2013). Plasmonic electrically functionalized TiO_2_ for high-performance organic solar cells. Adv. Funct. Mater..

[19] Liu Y.M., Sun Y., Rockett A. (2012). An improved algorithm for solving equations for intra-band tunneling current in heterojunction solar cells. Thin. Solid. Films..

[20] Yan H., Manion J.G., Yuan M.J., Arquer F.P.G.D., Seferos D.S. (2016). lncreasing polymer solar cell fill factor by trap-filling with F4-TCNQ at parts per thousand concentration. Adv. Mater..

[21] Liao H.H., Chen L.M., Xu Z., Li G., Yang Y. (2008). Highly efficient inverted polymer solar cell by low temperature annealing of Cs_2_CO_3_ interlayer. Appl. Phys. Lett..

[22] Olthof S., Mehraeen S., Mohapatra S.K., Barlow S., Coropceanu V., Bredas J.L., Marder S.R., Kahn A. (2012). Ultralow doping in organic semiconductors: Evidence of trap filling. Phys. Rev. Lett..

[23] Xu R., Zhang K., Liu X., Jin Y., Jiang X.-F., Xu Q.-H., Huang F., Cao Y. (2018). Alkali salt-doped highly transparent and thickness-insensitive electron-transport layer for high-performance polymer solar cell. ACS Appl. Mater. Interfaces.

[24] Koster L.J.A., Smits E.C.P., Mihailetchi V.D., Blom P.W.M. (2005). Device model for the operation of polymer/fullerene bulk heterojunction solar cells. Phys. Rev. B.

[25] Mihailetchi V.D., Xie H.X., Boer B.D., Koster L.J.A., Blom P.W.M. (2006). Charge transport and photocurrent generation in poly(3-hexylthiophene): Methanofullerene bulk-heterojunction solar Cells. Adv. Funct. Mater..

[26] Wehenkel D.J., Koster L.J.A., Wienk M.M., Janssen R.A.J. (2012). Influence of injected charge carriers on photocurrents in polymer solar cells. Phys. Rev. B..

[27] Fonash S., Arch J., Cuifi J., Hou J., Howland W., McElheny P., Moquin A., Rogossky M., Rubinelli M., Tran T. (1997). A Manual for AMPS-1D for Windows 95/NT.

[28] Zhu H., Kalkan A.K., Hou J.Y., Fonash S.J. (1999). Applications of AMPS-1D for solar cell simulation. AIP Conf. Proc..

[29] The University of Illinois and Engineering Wiki Website. https://wiki.engr.illinois.edu/display/solarcellsim.

[30] Hall R.N. (1952). Electron-hole recombination in germanium. Phys. Rev..

[31] Shockley W., Read W.T. (1952). Statistics of the recombinations of holes and electrons. Phys. Rev..

[32] Sandberg O.J., Nyman M., Österbacka R. (2014). Effect of contacts in organic bulk heterojunction solar cells. Phys. Rev. Appl..

