# Supplementary material for: Numerical Simulation of S-Shaped Current–Voltage Curves Induced by Electron Traps in Inverted Organic Photovoltaics
Source: Int J Mol Sci. 2022 Feb 12;23(4):2039. doi: 10.3390/ijms23042039 (PMC8880258; doi:10.3390/ijms23042039)
Supplement: Supplementary file 1 [file ijms-23-02039-s001.zip › ijms-1555190-supplementary.pdf]

Supporting Information

Numerical Simulation of S-Shaped Current - Voltage Curves Induced by Electron Traps in Inverted Organic Photovoltaics

Shanglin Luo [1], Mingfang Huo [1], Qin Xue [1,*], and Guohua Xie [2,*]

[1] Department of Physical Science and Technology, Central China Normal University, Wuhan 430079, China
[2] Sauvage Center for Molecular Sciences, Hubei Key Lab on Organic and Polymeric Optoelectronics Materials, Department of Chemistry, Wuhan University, Wuhan 430072, China
* Correspondence: xueqin@mail.ccnu.edu.cn (Q.X.) and guohua.xie@whu.edu.cn (G.X.);

List of physical parameters and symbols.

Elementary charge: q

Potential barrier: φ

Permeability in free space: ε_0

Relative dielectric permeability: ε_r

Concentrations of trapped electrons: n_t

Concentrations of trapped holes: p_t

Concentration of the ionized donors: N_D^+

Concentration of the ionized acceptors: N_A^-

Diffusion coefficients of electrons: D_n

Diffusion coefficients of holes: D_p

Electrical field: F

Electron current density: J_n

Hole current density: J_p

Generation rates of free charge carriers: G

Recombination rates of free charge carriers: R

Effective energy offset inside the active layer: E_{gap}

Intrinsic density of states at the conduction band: N_c

Intrinsic density of states at the valence band: N_v

Boltzmann constant: k

Absolute temperature: T

Electron mobility: μ_n

Hole mobility: μ_p

Surface recombination velocity of electrons (cathode side): $S_{n,cat}$

Surface recombination velocity of holes (cathode side): $S_{p,cat}$

Surface recombination velocity of electrons (anode side): $S_{n,an}$

Surface recombination velocity of holes (anode side): $S_{p,an}$

Energy level of the lowest unoccupied molecular orbital: E_{LUMO}

Energy level of the highest occupied molecular orbital: E_{HOMO}

Quasi-Fermi level for electrons: E_{Fn}

Quasi-Fermi level for holes: E_{Fp}

Open-circuit voltage: V_{OC}

Short-circuit current: J_{SC}

Thickness of the active layer: d

Thickness of the negatively charged region: d_c

Effective electron injection barrier: $\varphi_{1,eff}$

Effective built-in voltage: $V_{bi,eff}$

Energy offset: Δ

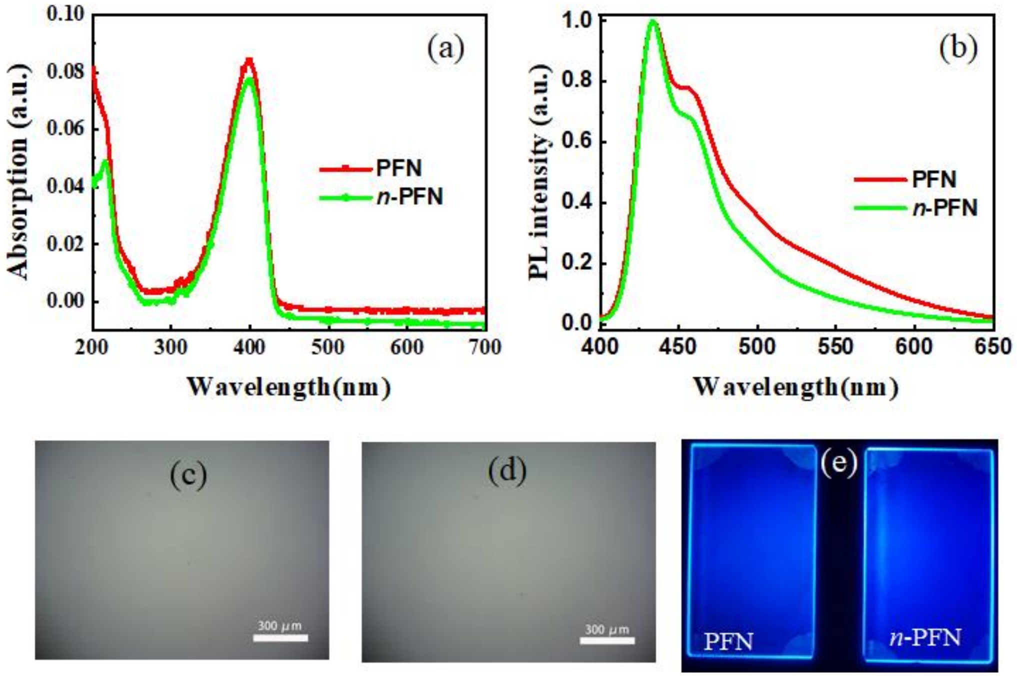

Figure S1. Absorption (a) and steady-state photoluminescence (b) of PFN and PFN:Cs_2CO_3 (5:1) spin-coated on quartz substrates. (c) PFN and (d) PFN:Cs_2CO_3 films captured by a microscope. (e) Photos of the films under UV 365 nm.

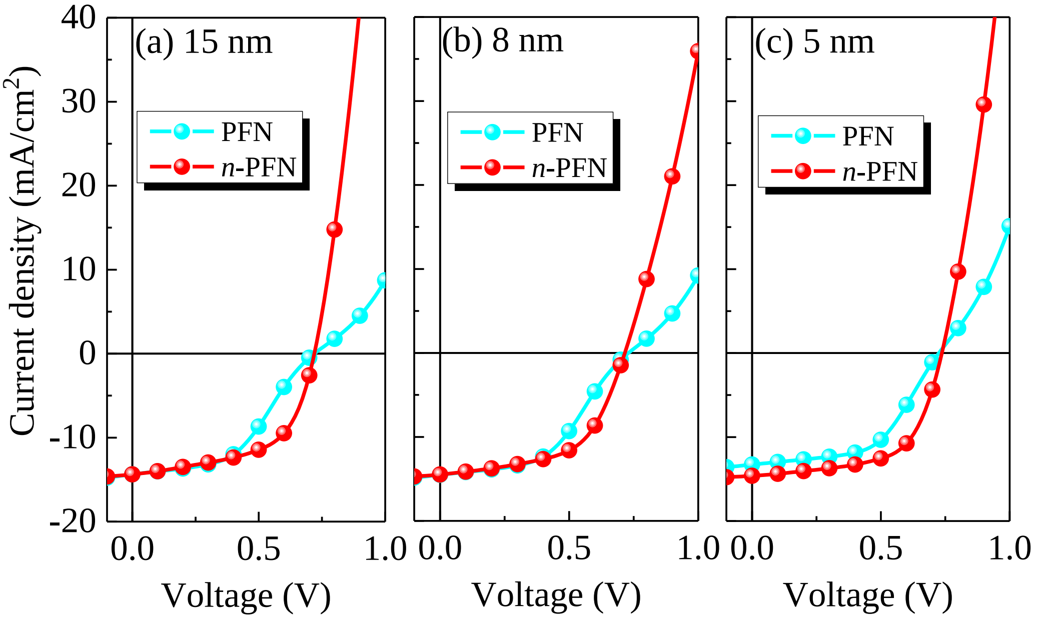

Figure S2. *J-V* curves of the devices with different thickness of the electron extraction layer: (a) 15 nm, (b) 8 nm, and (c) 5 nm.

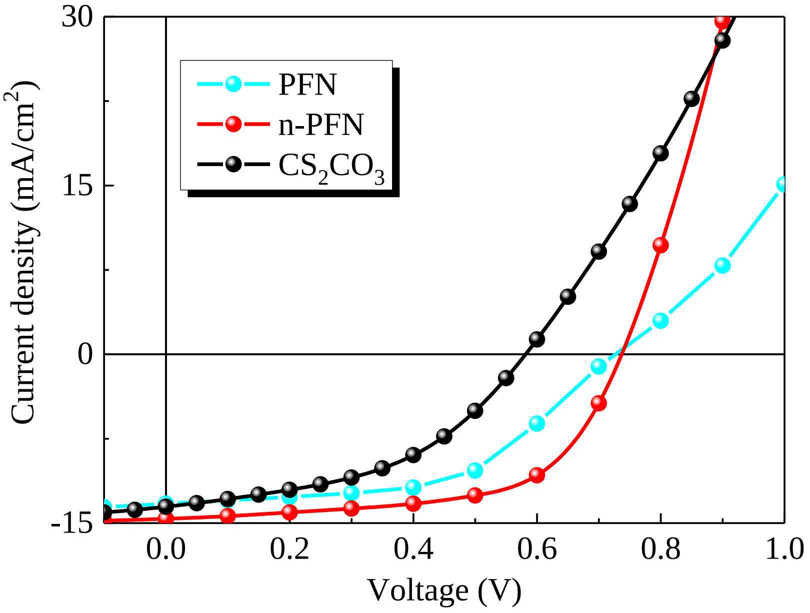

Figure S3. J-V curves of the devices with PFN (5 nm), PFN:Cs_2CO_3 (5 nm), and Cs_2CO_3 (1 nm).
